# Group 3 Innate Lymphoid Cells: Communications Hubs of the Intestinal Immune System

**DOI:** 10.3389/fimmu.2017.01298

**Published:** 2017-10-16

**Authors:** David R. Withers, Matthew R. Hepworth

**Affiliations:** ^1^College of Medical and Dental Sciences, Institute of Immunology and Immunotherapy (III), University of Birmingham, Birmingham, United Kingdom; ^2^Manchester Collaborative Centre for Inflammation Research (MCCIR), Division of Infection, Immunity and Respiratory Medicine, Faculty of Biology, Medicine and Health, School of Biological Sciences, Manchester Academic Health Science Centre, University of Manchester, Manchester, United Kingdom

**Keywords:** innate lymphoid cells, group 3 innate lymphoid cell, intestinal inflammation, interleukin-22, inflammatory bowel disease

## Abstract

The maintenance of mammalian health requires the generation of appropriate immune responses against a broad range of environmental and microbial challenges, which are continually encountered at barrier tissue sites including the skin, lung, and gastrointestinal tract. Dysregulated barrier immune responses result in inflammation, both locally and systemically in peripheral organs. Group 3 innate lymphoid cells (ILC3) are constitutively present at barrier sites and appear to be highly specialized in their ability to sense a range of environmental and host-derived signals. Under homeostatic conditions, ILC3 respond to local cues to maintain tissue homeostasis and restrict inflammatory responses. In contrast, perturbations in the tissue microenvironment resulting from disease, infection, or tissue damage can drive dysregulated pro-inflammatory ILC3 responses and contribute to immunopathology. The tone of the ILC3 response is dictated by a balance of “exogenous” signals, such as dietary metabolites and commensal microbes, and “endogenous” host-derived signals from stromal cells, immune cells, and the nervous system. ILC3 must therefore have the capacity to simultaneously integrate a wide array of complex and dynamic inputs in order to regulate barrier function and tissue health. In this review, we discuss the concept of ILC3 as a “communications hub” in the intestinal tract and associated lymphoid tissues and address the variety of signals, derived from multiple biological systems, which are interpreted by ILC3 to modulate the release of downstream effector molecules and regulate cell–cell crosstalk. Successful integration of environmental cues by ILC3 and downstream propagation to the broader immune system is required to maintain a tolerogenic and anti-inflammatory tone and reinforce barrier function, whereas dysregulation of ILC3 responses can contribute to the onset or progression of clinically relevant chronic inflammatory diseases.

## Background

Innate lymphoid cells (ILCs) constitute a family of tissue-resident innate lymphocytes with increasingly appreciated roles in tissue homeostasis, immunity, and inflammation (1–5). A rapidly developing body of evidence derived from mouse and human studies has begun to demonstrate how ILCs play critical, non-redundant roles in maintaining tissue health or in driving disease pathology. ILCs possess several characteristics that make them particularly suited to rapidly respond to perturbations in tissue homeostasis, infection, or tissue damage including (i) constitutive presence in barrier tissues and lymphoid organs, (ii) a “poised” transcriptional and epigenetic landscape, and (iii) the ability to respond rapidly and robustly to signals in the tissue microenvironment.

As reviewed extensively elsewhere (1–5), ILCs can be subdivided into groups on the basis of transcription factor expression and cytokine secretion profile. In this review, we will focus on group 3 innate lymphoid cells (ILC3), which are characterized by the expression of the transcription factor retinoic acid (RA)-related orphan receptor γ isoform t (RORγt) and the capacity to produce the cytokines interleukin (IL)-17A, IL-17F, IL-22, and GM-CSF. ILC3 differ from other ILC groups in that they constitute at least two bona fide subsets that are transcriptionally, developmentally, and functionally distinct and inhabit distinct tissue microenvironments [reviewed in Ref. (6)]. In mice, these subsets are distinguished by surface expression of natural cytotoxity receptors (NCR), particularly NKp46 (termed NCR^+^ ILC3), and molecules and receptors historically associated with fetal lymphoid tissue inducer (LTi) cells, such as lymphotoxin and the chemokine receptor CCR6 (termed LTi-like ILC3) (5, 6). ILC3 are found in a range of tissues and organs, most notably the gastrointestinal tract and associated lymphoid tissues, although the relative distribution of ILC3 subsets differs dependent upon tissue location. Indeed, while NCR^+^ ILC3 are the most prevalent ILC3 subset in the small intestine, LTi-like ILC3 appear to dominate in the colon and lymphoid tissues. Importantly, emerging evidence suggests that these two subsets also play distinct functional roles that relate to tissue-specific biological challenges.

Tissue-resident ILC3 sense and respond to a wide range of environmental and host-derived signals within the local microenvironment and integrate these cues to modulate cell-intrinsic transcription and to relay information to other cells, either through the production of cytokines or through cell–cell interactions. Indeed, as discussed below, ILC3 concurrently sense a multitude of soluble signals and environmental cues, which may change dynamically following infection or tissue damage, thus posing the question as to how ILC3 integrate and interpret these signals to respond appropriately. The balance of ILC3 effector functions may set the immunological tone of the tissue and help orchestrate the wider immune response. Although other ILC subsets also share the capacity to respond to a wide range of cues within the tissue microenvironment [reviewed in detail elsewhere—(4, 5, 7)], here, we will discuss the concept of ILC3 as tissue-resident sentinels and key “communications hubs” of the intestinal immune response.

## Communicating with the Outside World: ILC3 as Early Colonizers and Sentinels of Barrier Tissues

ILC3 are derived from fetal liver progenitors during embryogenesis and are among the first lymphocytes to seed barrier tissues, in particular the intestinal tract, prior to birth (8, 9). In this context, ILC3 are among the first-responders to colonization by commensal microbes, as well as diet-derived antigens introduced following weaning. Furthermore, these cells are central organizers of secondary lymphoid tissue organogenesis (10). ILC3 are therefore in prime position to shape the emerging mucosal immune system. Developmentally both ILC3 subsets derive from a common lymphoid progenitor ancestor; however, recent evidence has highlighted a divergent developmental relationship between ILC3 subsets and other ILC family members. The development of the wider ILC family is mediated through a series of transcriptional decisions that generate distinct progenitor populations with increasing commitment toward the ILC lineage, as reviewed extensively elsewhere (3, 11, 12). A key stage in this development is the bifurcation of the “cytotoxic” ILC lineage (i.e., classical NK cells), from the remaining “helper” ILC lineage (ILC1, ILC2, and ILC3). This is characterized by the development of a common helper ILC progenitor (CHILP) that expresses high levels of the transcription factor ID2 and IL-7R and which is able to generate ILC1, ILC2, and both ILC3 subsets but has lost the potential to generate progeny from other lymphocyte lineages (13). Additionally, a subset of ID2^+^ progenitors develops downstream of the CHILP, characterized by the expression of the transcription factor PLZF and surface PD-1—termed the common ILC precursor (ILCp) (14, 15). However, while ILCp can give rise to ILC1, ILC2, and NCR^+^ ILC3, they are unable to generate LTi-like ILC3 progeny (14), suggesting that a bifurcation of ILC3 subset development occurs during the progression from CHILP to ILCp. In line with these findings, LTi-like ILC3 have been demonstrated to derive from an ID2^+^ α_4_β_7_^+^ CXCR5^+^ progenitor cell that diverges upstream of ILCp, prior to the acquisition of PLZF (16–18). As such LTi-like ILC3 develop *via* a distinct route from the remaining ILC family members, including NCR^+^ ILC3, which may underlie key transcriptional and functional differences recently highlighted between ILC3 subsets (6, 19–21). Moreover, recent studies have uncovered plasticity among ILC3 and other ILC populations that is dictated by changes in the cytokine milieu within the tissue microenvironment (22–24). Thus, in addition to transcriptional decisions made during development, plasticity of mature ILC3 may shape the composition of these cells in tissues.

**Figure 1:**
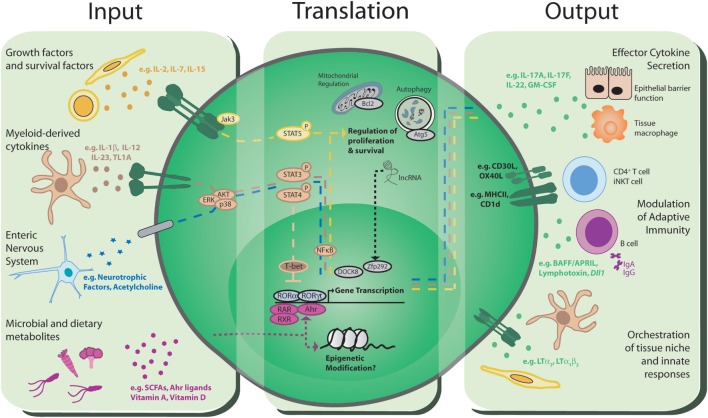
Group 3 innate lymphoid cells (ILC3): communications hubs of the intestinal immune system. *Input*: ILC3 have the capacity to receive environmental cues and host-derived signals from a range of sources including the immune system, stroma, the enteric nervous system, the microbiota, and the diet. *Translation*: these signals are integrated through a range of intracellular signaling mechanisms, including phosphorylation of STAT proteins, ligand-binding transcription factors, epigenetic modification, and multiple intracellular processes with the potential to support long-term survival of ILC3. Integration of multiple signals likely happens simultaneously, indicating a complicated and dynamic process that determines the threshold, magnitude, and type of ILC3 responses that are propagated to communicate with other cells in the intestinal environment. *Output*: translated signals are conveyed to other cells within the local environment through a variety of distinct mechanisms, most notably *via* the production of effector cytokines and growth factors that modulate and orchestrate the wider immune response and intestinal barrier function. The nature and balance of the signals perceived by ILC3 ultimately determine the downstream effector response, and dysregulation of homeostatic signals in disease may dramatically alter ILC3 responses and drive pro-inflammatory phenotypes. Finally, direct cell–cell communication through antigen presentation and co-stimulatory molecules can directly regulate the adaptive immune response.

Seeding of intestinal tissues and associated lymphoid structures by ILC3 occurs during embryogenesis and is further regulated by environmental signals encountered following birth. Seminal studies demonstrated LTi-like ILC3 are present in the fetal gut, whereas NKp46^+^ ILC3 are largely absent but proliferate rapidly following birth to become the dominant ILC3 subset in the small intestine (8). ILC3 subset maturation and migration to the gut is in part dictated by maternal, microbial and dietary signals (Figure 1; Inputs). The extent to which ILC3 can directly sense microbial-derived cues remains poorly understood. Indeed, unlike many myeloid cell populations, ILC3 do not appear to express toll-like receptors or other canonical pattern recognition receptors. Rather ILC3 responses to microbial cues are dependent upon other key intermediaries, such as resident mononuclear phagocyte (MNP) populations that convey information to ILC3 (detailed below). ILC3 are particularly sensitive to alterations in the microbiota and their numbers are modulated following neonatal colonization by commensal microbes, in part through an IL-25-dependent negative feedback mechanism that restricts expansion of ILC3 in a microbiota-dependent manner (9). Recent studies suggest that ILC3 are directly regulated by bacterial metabolites, such as short-chain fatty acids (SCFAs) produced through microbial metabolism of dietary fiber. Consistent with this, NCR^+^ ILC3 present in the Peyer’s patches (PP) of the ileum were found to express *Gpr109a*—the receptor for butyrate—and SCFA signaling acted to restrict NCR^+^ ILC3 numbers and cytokine production (25). Additionally, NCR^+^ ILC3 are dependent upon the aryl hydrocarbon receptor (Ahr) for their development and persistence in the small intestine (26–29). Ahr ligands can be derived from intestinal bacteria, as well as dietary and endogenous sources and further regulate cytokine production of mature adult ILC3, while immunoglobulin-bound Ahr ligands are also transferred from the mother to promote the migration of NCR^+^ ILC3 to the neonatal small intestine (30).

In addition to microbial metabolites, ILC3 are subjected to further direct modulation by dietary cues. Maternal retinoids transferred during embryogenesis favor the development and maturation of LTi-like ILC3 by promoting and stabilizing RORγt expression (31), while dietary vitamin A-derived RA is required for the maintenance of adult small intestinal ILC3 subsets (32–34). In support of a role for dietary vitamins in the regulation of ILC3 responses, human ILC3 were found to up-regulate vitamin D receptor expression following cytokine stimulation and vitamin D treatment subsequently resulted in suppression of the IL-23R, implicating vitamin D as a regulator of ILC3 function (35). In line with this vitamin D receptor-deficient mice exhibit elevated ILC3 numbers and IL-22 production (36). Taken together, these studies implicate dietary vitamins as key modulators of ILC3 numbers and function and demonstrate the capacity of ILC3 to directly sense dietary signals.

## Setting the Tone: Homeostatic Inputs Maintain Adult ILC3 Subsets

In addition to environmental cues derived from the microbiota and diet, the numbers and relative balance of ILC3 subsets at steady state are additionally regulated by host-derived inputs (Figure 1; Inputs). In contrast to the dynamic changes observed within the neonatal intestine, adult ILC3 populations appear to be stable and long-lived with little to no replenishment from the bone marrow under homeostatic conditions (37). Current understanding suggests that ILC3 are maintained through self-renewal and/or replenished from tissue-resident precursors and several groups have reported the presence of tissue-resident RORγt^+^ progenitors that can give rise to mature ILC3 subsets in both mice and humans (38, 39). In particular, intestinal resident NKp46^−^CCR6^−^RORγt^+^ ILC3 precursors give rise to NCR^+^ ILC3, and to a lesser extent the LTi-like ILC3 compartment, a process that is regulated by local tissue cytokines and Notch signaling (40–44). Adult ILC3 subsets differ in their tissue localization; CCR6^+^ ILC3 are largely restricted to lymphoid structures such as the mesenteric lymph node and cryptopatches and ILFs within the small intestine and colon (45, 46), while NCR^+^ ILC3 are largely excluded from lymph nodes and colon but reside within both the lamina propria and ILFs of the small intestine, with localization of NCR^+^ ILC3 dictated in part through CXCR6 expression (47–51). Thus, local signals perceived by ILC3 subsets likely dictate their maintenance and survival within specific tissue microenvironments.

While the cell-extrinsic and cell-intrinsic signals that support ILC3 under homeostatic conditions remain incompletely defined, recent evidence suggests that ILC3 subsets are differentially dependent on constitutive signals received *via* pro-survival cytokines for their maintenance. ILC3 constitutively express high levels of common γ chain cytokine family receptors including IL-2R, IL-7R, and IL-15R. Of these, the IL-7:IL-7R interaction has been most extensively studied and IL-7 deficient mice have reduced numbers of lymph nodes, consistent with a loss of LTi cells in the embryo, and decreased ILC3 numbers in the adult (40, 52–54). Furthermore, enhanced signaling through IL-7Rα increases LTi-like cell numbers in the adult (53), arguing that IL-7 has the capacity to directly regulate ILC3 numbers. Notably overexpression of TSLP, which signals through a heterodimer of TSLPR and IL-7R, could overcome the effect of IL-7 deficiency, further arguing that other signals can compensate (55). A more recent study demonstrated that residual numbers of all ILC groups persist in the absence of IL-7, with IL-15 necessary to support the survival of the remaining NCR^+^ ILC3, but not LTi-like ILC3, in the intestinal tract (56). Provision of survival signals such as IL-7, IL-15, and TSLP is mediated largely through critical interactions with non-hematopoietic, stromal cell populations (Figure 1; Inputs). Studies of IL-7 reporter mice identified marginal reticular cells as a key source of this cytokine in lymph nodes (57), although analysis of IL-7 mRNA indicates that fibroblastic reticular cells (FRCs) also produce IL-7 (58). Strikingly, production of IL-7 by stromal cells requires signaling through the lymphotoxin β-receptor (59), indicating that continued interactions between stroma and lymphotoxin expressing immune cells support normal FRC function, which in turn maintains ILC3. Similarly, FRCs in lymph nodes and PP are a key source of IL-15, which supports NCR^+^ ILC3 as well as NK cells and ILC1 (56, 60). Thus, the stromal infrastructure of tissue microenvironments provides much of the survival requirements of ILC3. However, there is evidence that some LTi-like ILC3 persist in the absence of both of IL-7 and IL-15 (56), arguing for a role for other cytokine signals or indicating that alternative mechanisms of survival exist. Given that LTi-like ILC3 also highly express CD25 (IL-2R) and are enriched in lymphoid structures in close proximity to proliferating T cells (61, 62), it is possible that IL-2 may play an additional role in the long-term maintenance of LTi-like ILC3. Together these findings suggest that stromal cells form critical niches within secondary and tertiary lymphoid tissues that provide signals required to support ILC3 survival.

## Talking to Yourself: Diverse Host-Derived Signals Regulate ILC3 Effector Responses

As addressed earlier, ILC3 have the capacity to receive input from cells and soluble molecules within their local tissue microenvironment (Figure 1; Inputs). In addition to stromal cells, other innate immune cells, such as MNPs, play key roles in both homeostatic and effector ILC3 responses (63, 64). Within the intestine it is clear that IL-23, produced by CX_3_CR1^+^ MNPs, is a key regulator of ILC3 function (65–67). CX_3_CR1^+^ MNPs were observed to cluster with ILC3 in distinct intestinal lymphoid tissues, such as cryptopatches. Following weaning sensing of the microbiota by intestinal lymphoid tissue, MNPs result in local production of IL-23 and the induction of IL-22 from lymphoid tissue-resident LTi-like ILC3 (46). Moreover, depletion of CX_3_CR1^+^ MNPs results in a failure to control *Citrobacter rodentium* infection due to impaired IL-22 production by ILC3. In addition to IL-23, IL-1β drives IL-22 production by ILC3 and this is further augmented by TL1A, again produced by CX_3_CR1^+^ MNPs (67). The balance of cytokine signals perceived by ILC3 is also critical in determining phenotype and function. Indeed, intestinal ILC3 exhibit significant plasticity and signals including IL-12, IL-15, and IL-18 released in the context of infection and inflammation promote the progressive up-regulation of T-bet and production of pro-inflammatory cytokines such as IFN-γ and TNF-α by NCR^+^ ILC3 and a subsequent loss of RORγt expression by this subset, resulting in these cells being labeled “ex-ILC3” (40, 41). Conversely, upon resolution of infection or inflammation, restoration of homeostatic levels of MNP-derived signals including IL-23 and IL-1β favors the reconversion of inflammatory “ex-ILC3” back to an RORγt^+^ NCR^+^ ILC3 phenotype (43, 68).

ILC3 may also respond to other fundamental innate immune factors, such as the complement system. A subset of ILC3 appear to be sensitive to the complement cascade *via* expression of the C3aR (69), while Complement Factor P—a positive regulator of the alternative complement pathway—was found to directly bind NKp46 (70), suggesting NCR^+^ ILC3 in particular may sense pathogen infection *via* interactions with the complement cascade. Beyond interactions with the immune system, an increasing body of evidence indicates intestinal resident ILC3 can directly sense cues from the enteric nervous system. In a series of studies, LTi-like ILC3 were demonstrated to express RET—a receptor for neurotrophic factors (71, 72). RET expression is required for PP formation and accumulation of lymphotoxin-producing LTi cells, although this occurs indirectly *via* ligation of RET on lymphoid tissue-initiator cells and induction of chemokine production to sequester immune cells, including LTi cells, to drive PP formation. Nonetheless, adult CCR6^+^ LTi-like ILC3 express RET and can directly respond to glial-derived neurotrophic factor (GDNF) family ligands. Production of GDNF by intestinal glial cells in response to stimulation by microbial ligands acts to reinforce intestinal barrier function *via* regulation of IL-22 transcription and secretion by LTi-like ILC3 (72). Interestingly, enteric neurons also have the capacity to provide RA signals, which are critical for the maturation of LTi cells (73), thus suggesting that alternative, non-dietary sources of RA may also play roles in ILC function and immune homeostasis. In addition to local neuronal signals, systemic nervous signals may modulate the tone and magnitude of ILC3 responses. Vagal nerve innervation of the colon is required for the formation of tertiary lymphoid structures, *via* regulation of local chemokine production (74). Although a direct effect of vagal denervation on ILC3 was not demonstrated in this study, more recent evidence suggests that the vagal nerve acts to regulate ILC3 responses to bacterial pathogens in the peritoneal cavity (75). This effect was in part dependent upon the ability of NCR^+^ ILC3 to enzymatically generate lipid precursors that were in turn metabolized by resident macrophages to promote resolution of inflammation (75). Together these findings suggest signals from both tissue-resident and systemic neurons may directly regulate ILC3 numbers and function during homeostasis or following infection. Interestingly, ILC3 may also play important roles in the central nervous system and a recent study demonstrated that NCR^+^ ILC3 are present in the meninges and promote neuroinflammation in a model of multple sclerosis by licensing entry of inflammatory Th17 cells into the brain (76). It is highly likely that other signals from the nervous system and beyond may impact upon ILC3 function. For example, host-derived lipid mediators—such as prostaglandin E_2_—have been demonstrated to directly promote homeostatic ILC3 cytokine production (77). Similarly, endocrine signals, particularly sex hormones, are long appreciated to regulate innate immunity. In this regard testosterone directly inhibits ILC2 responses (78); however, the capacity of androgens, estrogens, or other hormones to modulate ILC3 is yet to be investigated.

## Translating the Message: Integration and Transcriptional Dynamics

The ability of tissue ILC3 to sense a breadth of exogenous and endogenous cues of significantly varying natures provokes the question as to how these various inputs are simultaneously integrated, and prioritized, in order to control ILC3 function. While significant advances have been made in understanding the effector functions of ILC3, as well as the transcriptional decisions that determine ILC development, relatively little is known about the signaling pathways and transcriptional dynamics that act to integrate external cues and determine the nature and magnitude of downstream ILC3 responses. Advances in RNA sequencing and epigenetic profiling have begun to redress this balance and allowed for enhanced resolution of individual cellular states among ILC populations (18–21, 79, 80). Furthermore, comparison of the transcriptional networks of ILC3 subsets and their Th17 counterparts will likely prove useful in identifying common and distinct signaling pathways utilized by RORγt-expressing cells (81). While significant overlap may be expected in this case, it is critical to note that ILC3 and Th17 cells also demonstrate key difference in their transcriptional regulation. For example, while Th17 is acutely dependent on RORγt for the maintenance of a mature functional phenotype, ILC3 were able to maintain core effector functions and phenotype following deletion of RORγt (82). Surprisingly, human IL-22-producing ILC3 can be generated from circulating ILCps even when derived from *Rorc*-deficient donors (83). Thus, it is likely that ILC3 also exhibit unique and differing signaling transduction pathways and transcriptional regulation that underlie their innate functions (Figure 1; Translation). As detailed earlier, ILC3 transcription dynamics are acutely modified by dietary and bacterial metabolites *via* Ahr and it is likely epigenetic changes may be imprinted *via* histone deacetylases downstream of SCFA-sensing, as in other lymphocytes (84).

ILC3 additionally integrate multiple soluble signals including common gamma chain cytokines and growth factors—many of which induce signal transduction *via* phosphorylation of STAT5 (85). Thus, pSTAT5 and downstream signal activation (ERK/AKT) are likely to play key roles in orchestrating intracellular responses in ILC3. Similarly, both IL-23 and RET signals are transduced in part by pSTAT3 to regulate IL-22 production (72, 86). Thus, it is likely that a threshold of intracellular signaling downstream of multiple receptors, sensing cues from multiple biological systems, acts to establish the tone and magnitude of the ILC3 response (Figure 1; Translation). The signaling pathway engaged following stimulation by cytokines or other cues is likely to determine the biological processes that are modulated. For example, ILC3 from mice with mutations in *Jak* signaling exhibited an impaired ability to phosphorylate STAT5 in response to IL-7, while inhibiting *Jak3* signaling in mature human ILC3 suppressed proliferation of these cells but not cytokine production (87).

Maintenance of ILC3 subsets is likely to be regulated transcriptionally *via* multiple mechanisms (Figure 1; Translation). Mice lacking the scaffolding protein dedicator of cytokinesis 8 (DOCK8) demonstrate reduced ILC3 numbers and defective immunity to *C. rodentium*, in part due to a reduced ability of DOCK8-deficient ILC3 to respond to IL-7 and IL-23 and an increased rate of apoptosis (88). Similarly, survival of ILC3 is modulated *via* long non-coding RNAs that orchestrate downstream gene accessibility (89). In particular, *lncKdm2b* expression by ILC3 controls activation of transcription factors, including *Zfp292*, and recruitment of chromatin organizational machinery that promote ILC3 maintenance in vivo (89). ILC3 survival may also be dependent upon expression of anti-apoptotic machinery. Indeed, LTi-like ILC3 highly express the anti-apoptotic molecule Bcl-2, expression of which fluctuates following perturbation of local cytokine signals such as IL-7 (56). In addition, regulation of intracellular organelle degradation through autophagy has been implicated in ILC survival (90). The autophagy related factor *Atg5* was found to be required for ILC family development, suggesting that autophagy may play a role in ILC3 persistence and maintenance (90). Nonetheless, and despite these advances, many of the precise mechanisms through which ILC3 maintenance is instructed *via* changes in gene expression or epigenetics and the environmental signals that are required to promote ILC3 survival are yet to be defined.

## Pass it On: ILC3 Orchestration of Intestinal Tissue Immune Responses

ILC3 possess multiple mechanisms through which they relay host and environmental signals to orchestrate the intestinal immune response and to maintain tissue function (Figure 1; Outputs). ILC3 were initially identified as potent sources of effector cytokines, most notably IL-22. ILC3-derived IL-22 plays critical roles in regulating host-commensal bacteria interactions in the healthy intestine in addition to mediating responses to enteric pathogen infections (27, 86, 91–98). IL-22 mediates its effects on IL-22R-expressing non-hematopoietic cells, including intestinal epithelial cells, epithelia-associated stem cells, and Paneth cells (99–101), and promotes barrier function and segregation of commensal bacteria from the underlying immune system *via* induction of epithelial tight junction proteins (97, 102), fucosylation of epithelial cell-associated glycans (103–105), secretion of mucin, and production of anti-microbial peptides (e.g., S100A8/A9 and RegIIIβ/γ), which together induce bacterial killing and prevent translocation of commensal organisms into the circulation and peripheral organs (102).

Although the importance of ILC3 in regulating intestinal health and inflammation has largely been ascribed to the production of IL-22, ILC3 produce several other cytokines that contribute to intestinal immune responses. In particular, ILC3 subsets have the capacity to produce IL-17A and IL-17F (2). Although both embryonic LTi and adult ILC3 isolated from the small intestine can be induced to secrete IL-17A upon ex vivo stimulation (9), fate mapping of IL-17A producing cells revealed that only a small proportion of ILC3 in the intestine exhibit a history of IL-17A expression (106). Nonetheless, ILC3-derived IL-17A may contribute to the formation of pulmonary tertiary lymphoid structures following infection and inflammation (107), can contribute to host immunity to fungal and bacterial pathogens (63, 108, 109), and has been implicated in the pathogenesis of obesity-associated airway hyper reactivity (110). Additionally, dysregulated IL-17A production by ILC3 may act to exacerbate inflammation and disease pathology in a range of inflammatory diseases, including psoriasis and IBD (65, 111, 112). IL-17F has high homology to IL-17A and can be secreted as either a homodimer or heterodimer with IL-17A (113). ILC3 are a dominant source of IL-17F after induction of colitis in *Rag*1^−/−^ mice (65, 114), upon oral infection with *Candida albicans* and following skin wounding (108, 115), suggesting that IL-17F production by ILC3 may play critical roles in inflammation and immunity or resolving immune responses. Although IL-17F has largely been ascribed pro-inflammatory roles and may play pathogenic roles in colitis models (116, 117), it can also synergize with IL-22 to enhance production of anti-microbial peptides (118). Despite this evidence, the exact role of ILC3-derived IL-17F and its mechanisms of action in infection and disease, and how its effects differ from those of IL-17A, remain incompletely understood.

Recent studies have additionally highlighted ILC3 as a potent source of the cytokine and growth factor GM-CSF (67, 119–121). GM-CSF modulates myelopoiesis in the bone marrow, as well as extramedullary hematopoiesis in tissues, and acts on mature peripheral myeloid cells including monocytes, macrophages and neutrophils by regulating their activation, maturation, and migration into tissues (122). ILC3 are the predominant source of GM-CSF at steady state in the intestinal tract, with both NCR^+^ and LTi-like ILC3 capable of GM-CSF secretion. Under homeostatic conditions ILC3-derived GM-CSF acts to maintain immune tolerance by regulating DC subsets that further promote regulatory T-cell populations (120). Thus, constitutive homeostatic ILC3-derived GM-CSF secretion acts to maintain a tolerogenic environment. The interplay between MNPs and ILC3 in the intestine is bidirectional. Indeed, microbiota-dependent signals, including IL-1β, IL-23, and TL1A, derived from intestinal MNPs act to potentiate GM-CSF production by ILC3 (67, 120), suggesting the potential for a regulatory feedback loop in the intestine regulated by ILC3-derived GM-CSF-dependent crosstalk with myeloid cells. This crosstalk may also be important in the context of intestinal inflammation and following perturbation of intestinal barrier function as MNP-derived cytokines were found to regulate ILC3 production of IL-22, in addition to GM-CSF, in mouse models of colitis and human IBD patients (67). In contrast to a tissue-protective role for ILC3-derived GM-CSF two independent studies demonstrated that ILC3-derived GM-CSF acted to exacerbate intestinal pathology in an innate cell driven model of colitis (anti-CD40 treatment of *Rag1*^−/−^ mice), in part through recruitment of inflammatory monocytes (121, 123). Furthermore, onset of colitis results in migration of ILC3 out of intestinal cryptopatches and into the lamina propria in a GM-CSF-dependent manner (121), further demonstrating that GM-CSF-dependent ILC3-MNP crosstalk may dictate the migration and localization of immune populations within the intestinal tissue.

## Regulation of Adaptive Immune Responses

Embryonic LTi cells are required to generate secondary lymphoid tissues, and this role has been expertly reviewed before (10, 124) Further to this role in establishing the microenvironments that foster B- and T-cell responses, more recent studies have revealed that CCR6^+^ LTi-like ILC3 contribute to the regulation of adaptive immune responses *via* both indirect and direct interactions with the adaptive immune system (Figure 1; Outputs). CCR6^+^ ILC3 reside within the spleen, mucosal-associated lymphoid tissues, and lymph nodes—particularly those draining mucosal sites such as the mesenteric and mediastinal lymph nodes (62). Phenotypically, adult LTi-like CCR6^+^ ILC3 are very similar to the embryonic LTi population but additionally express molecules such as OX40L and CD30L that may foster interactions with lymphocytes (125–128). Interestingly, expression of OX40L can be induced in embryonic LTi cells through ex vivo culture with inflammatory cytokines, such as TL1A (129). Whether embryonic-derived LTi persist in the neonate and adult, and for how long, is unclear—but given the presence of long-lived LTi-like cells in the adult and the functional heterogeneity of ILC3 subsets, the potential persistence of bona fide embryonic LTi cells after birth needs to be addressed. It is striking that CCR6^+^ ILC3 in adult PP, LNs, and spleen associate with stromal populations that closely resemble the embryonic “organizer” cells through which fetal LTi orchestrate lymphorganogenesis (57, 130). Thus, tissue microenvironments fostered early in the life in secondary lymphoid tissues by ILC3 may be maintained in the adult. In support of this, restoration of the splenic white pulp architecture after viral infection was delayed in the absence of ILC3 (131).

Direct regulation of CD4^+^ T cells by ILC3 can be mediated through MHCII-dependent antigen presentation. ILC3-conditional deletion of MHCII resulted in moderate colitis due to a failure to control T-cell responses to commensal bacteria (61, 132). Thus, within the gastrointestinal tract, ILC3 appear to play a crucial suppressive role in regulating CD4 T-cell responses to commensal organisms to maintain tissue homeostasis. Mechanistically, ILC3 were found to control commensal bacteria-specific CD4^+^ T-cell responses in part by outcompeting T cells for IL-2, thus starving that T cells of growth factors needed for proliferation and resulting in apoptosis (61). Similarly, ILC3 may regulate the T-cell pool by controlling the availability of other critical growth cytokines in lymphoid tissues, including IL-7 (133). Further investigations have indicated that the outcome of ILC3:T-cell interactions may be determined by the specific tissue environment, with splenic ILC3 found to support, rather than suppress, CD4 T-cell responses in an MHCII-dependent manner (134). This discrepancy could be partially explained by the ability of cytokines to modulate ILC3—for example, IL-1β can induce ILC3 expression of co-stimulatory molecules (e.g., CD80 and CD86) and alter the nature of ILC3 antigen presentation (134). In addition to the presentation of protein-derived antigenic peptides *via* MHCII, LTi-like ILC3 have the capacity to present lipid antigens to iNKT cells *via* surface CD1d (135). Furthermore, ILC3 are required to suppress homeostatic CD8^+^ T-cell expansion in neonatal mice (136).

ILC3 also have the capacity to modulate humoral immunity (Figure 1; Outputs). ILC3 present in the spleen and PP support innate T-cell-independent IgA production through production of secreted and membrane bound lymphotoxin, which supports local DC populations and aids IgA class switching (137–139). Similarly, splenic ILC3 provide B-cell growth factors, including BAFF/APRIL and *Dll*1, to enhance local Ab production by marginal zone innate B cells (119). Despite these advances, the full extent and nature of the crosstalk between ILC3 and other lymphocyte populations, and how these signals are integrated alongside those provided by traditional antigen-presenting cell populations such as DC and B cells, remain to be fully elucidated.

## Lost in Translation: Dysregulated ILC3 Communication and Disease

Here, we have highlighted the roles of ILC3 in integrating signals from the environment and relaying information to surrounding immune and non-immune cells, thus functioning as a critical communications hub within intestinal tissue. Through being able to respond to both epithelial and myeloid-derived cytokines, vitamins, metabolites, and also neuropeptides, ILC3 integrate a wealth of regional cues to maintain the appropriate balance of key effector molecules and ensure local tissue homeostasis. Thus, while ILC3 have a clear protective role in the tissue, dysregulation or dramatic changes in environmental cues can result in disrupted ILC3 communication and may contribute to disease pathology, in part *via* altered ILC3 effector functions. For example, dysregulated cytokine production in the context of mouse models of colitis can promote ILC3 production of disease driving pro-inflammatory cytokines such as IFN-γ and IL-17A (40, 68, 111). Similarly, while ILC3-derived IL-22 is critical for supporting homeostatic intestinal barrier function, epithelial cell repair and regeneration, chronic overproduction of IL-22 by ILC3 may promote colorectal cancers (140, 141). Interestingly, genetic polymorphisms associated with chronic inflammatory disease or cancer may also alter the inflammatory milieu and have the potential to drive dysregulated ILC3 communication. For example, Card 9 deficiency results in disrupted IL-1β production and impacts upon epithelial cell proliferation and colitis-associated cancer due to perturbed ILC3-associated IL-22 production (142). It is likely that many other polymorphisms seen in patients with intestinal inflammatory disorders, including IL-23R, IL-10/IL-10R, *Atg16l1*, and *Nod2*, also impact upon ILC3 function either directly or by altering the integration of tissue-specific signals that are sensed, interpreted or propagated by ILC3. Dysregulation of protective ILC3 functions in intestinal disease is also not limited to effector cytokine production. Indeed, ILC3-intrinsic expression of MHCII has been observed to be reduced in two separate cohorts of Crohn’s patients and found to correlate with enhanced Th17 responses in disease (61, 143), suggesting that the altered intestinal tissue environment may impact upon antigen presentation by these cells.

Infections may additionally disrupt ILC3 populations in the gut, resulting in a loss of their normal sentinel activity, the breakdown of gut barrier integrity, and intestinal pathology. In particular, infection with human or simian immunodeficiency viruses (HIV/SIV) results in depletion of ILC3 from the intestinal mucosa and lymph nodes (144–147). Thus, further investigation is needed to understand how alterations in ILC-related signals result in loss of these cells and how this balance can be redressed to restore homeostatic numbers and functions of ILC3. Finally, as the next generation of anti-inflammatory therapeutics enter the clinics, their relative impact on beneficial intestinal ILC3 need to be thoroughly addressed. In particular, monoclonal antibodies and small molecule inhibitors targeting common pathways shared by Th17 and ILC3 (e.g., anti-IL-23, anti-IL-12, anti-IL-17A, and small molecule antagonists of RORγt) have the potential to suppress inflammation but may have long-term consequences for patients by disrupting protective ILC3 pathways. In this regard, an increased understanding of the differences and similarities between Th17 and ILC3 regulation is required to guide therapeutic interventions and treatment regimen. Promisingly, recent studies suggest that acute targeting of RORγt effectively reduces Th17-driven inflammation while leaving protective ILC3 responses intact (82), although chronic inhibition of this master transcription factor may eventually negatively impact upon ILC3 responses. Furthermore, ILC3-derived cytokines may have beneficial roles in maintaining epithelial barrier function and healthy host–microbe interactions, thus targeting that these cytokines and their receptors in the context of Th17-driven inflammation may result in undesirable consequences. Indeed, treatment with neutralizing monoclonal antibodies against IL-17A or its receptor has been reported to worsen disease and increase incidence of adverse effects in several IBD patient cohorts (148, 149), further highlighting the need to understand the potential repercussions of emerging therapeutics on ILC3. Future studies will lead to a further understanding of how these critical innate immune sentinels are regulated in order to harness their protective functions to maintain tissue health, while suppressing dysregulated responses that exacerbate disease.

## Author Contributions

This review was jointly written by both authors, who contributed equally.

## Conflict of Interest Statement

The authors declare that the research was conducted in the absence of any commercial or financial relationships that could be construed as a potential conflict of interest.
